# Compartment syndrome following a jellyfish sting: a case report

**DOI:** 10.1186/s13256-022-03714-y

**Published:** 2023-01-06

**Authors:** Mohamed Elkafafi, Hussein Hamed, Yaser Ali, Mohamed Elgohary

**Affiliations:** 1 Paediatric Intensive Care Unit, Paediatric Department, Burjeel Hospital, Abu Dhabi, United Arab Emirates; 2 Paediatric Surgery Department, Burjeel Hospital, Abu Dhabi, United Arab Emirates

**Keywords:** Jellyfish, Sting, Compartment syndrome, Decompression fasciotomy, Case report

## Abstract

**Background:**

While the majority of reported cases of jellyfish envenomation are self-limited with few lasting complications, a few can cause life-threatening and debilitating illnesses. We present the case of a 15-year-old male who had an unusual presentation of a jellyfish sting that led to acute compartment syndrome.

**Case presentation:**

A 15-year-old Lebanese (Arab) boy was stung by a jellyfish, which led to acute compartment syndrome in the left arm. Decompression fasciotomy and local application of diluted nitroglycerin helped to relieve the ulnar and radial artery spasms. The patient was left with shoulder and elbow pain and elbow flexion weakness, which improved after physiotherapy over a period of 6 weeks.

**Conclusions:**

Current therapy recommendations for acute compartment syndrome following jellyfish stings are mainly based on case reports. Urgent fasciotomy and local application of nitroglycerin have been demonstrated to be helpful in severe jellyfish stings associated with acute compartment syndrome.

## Introduction

Jellyfish envenomation (the injection of venom) is a common incident in coastal areas all over the world. While the majority of reported cases are self-limited with few lasting complications, the stings of a few deadly species of jellyfish can cause life-threatening and debilitating illnesses with a prolonged recovery time [1, 2].

Jellyfish (or jellies) are members of the phylum Cnidaria (Coelenterata). The jellyfish body consists of a gelatinous umbrella-shaped bell and trailing tentacles, and its sting contains toxic peptide, phospholipase A, and histamine-liberating factors [3, 4].

Jellyfish float in salt water and sting when they come in contact with human bodies. The toxic venom can result in a variety of symptoms, including pain, swelling, redness, and, rarely, severe systemic reactions. The immediate or delayed clinical signs are based on toxicological and immunological responses to components of the venom and barbed tubules, including proteinaceous porins, neurotoxic peptides, bioactive peptides, collagens, and chitins [5, 6]. There are no specific data from the United Arab Emirates (UAE), but very few cases have been reported from coastal regions [7].

More than 100 species of jellyfish are known to be dangerous to humans. The treatment of the stings depends on the severity of the illness, the size and type of the jellyfish, and the individual response of the patient [2, 8].

## Case report

**Fig. 1:**
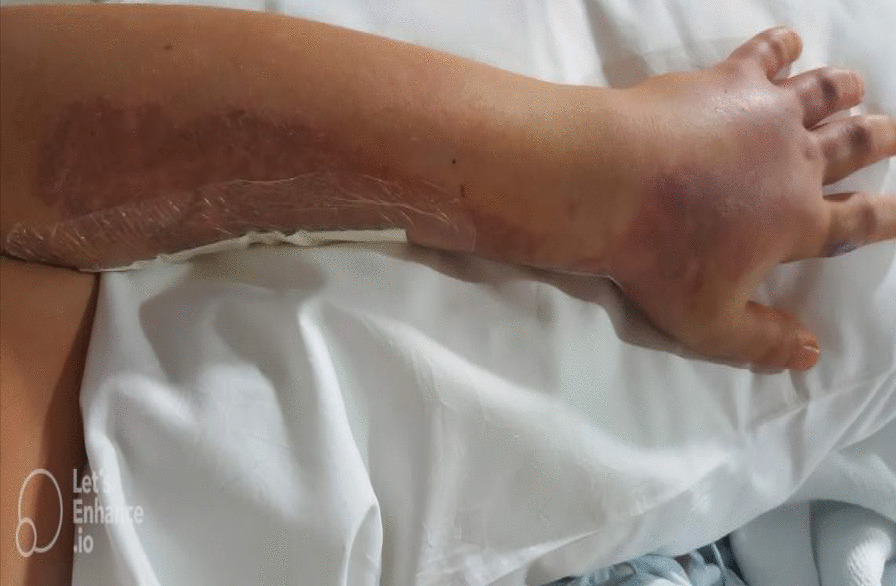
Postoperative appearance, with edema and ecchymotic patches over the arm, and dorsum of the hand and fingers

We report the case of a 15-year-old Lebanese (Arab) boy who was stung by a jellyfish on 27 September 2018 while on a beach of the Arabian Gulf. He was initially treated with pain medication and local creams; however, his condition deteriorated, with associated excruciating pain and forearm edema. He visited our pediatric emergency service 24 hours later with severe pain, forearm edema, and blue and cold fingers. On examination, the left forearm was swollen and very tense with cellulitis, and there was a large reddish patch on the dorsal aspect of the forearm where the sting occurred. The skin was muffled and cold compared with the right side. The capillary refill was sluggish. The child was able to actively extend the fingers (Fig. 1).

There was no palpable radial or ulnar pulse on the left side.

**Fig. 2:**
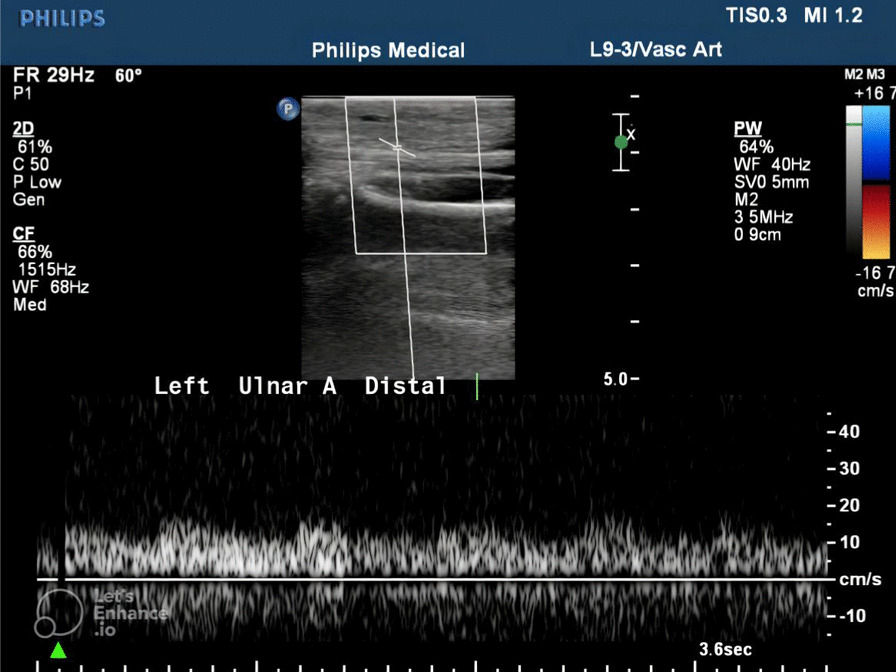
Reduced peak systolic velocity involving the left distal ulnar artery

**Fig. 3:**
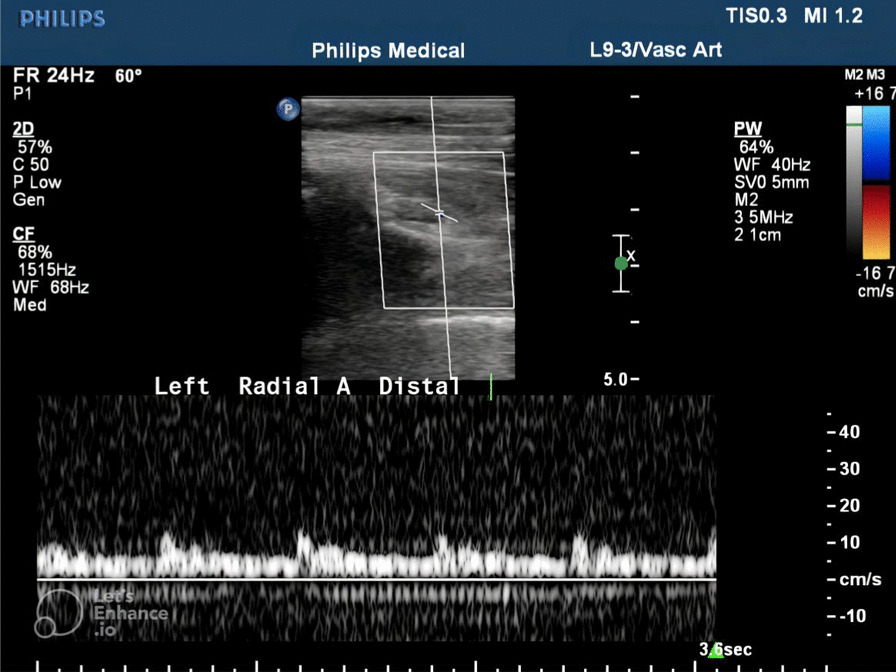
Reduced peak systolic velocity involving the left distal radial artery

The brachial artery showed a good signal on a portable Doppler scan, but no signal was retrieved at the level of the radial and ulnar arteries (Figs. 2 and 3).

The main concern was compartment syndrome with the occlusion of the arterial blood supply, which could affect the function of the arm and hand. Informed consent was obtained. Decompression fasciotomy was performed by making a curvilinear incision at the volar aspect of the left forearm. The subcutaneous tissues were dissected. The superficial and deep compartments were both incised and explored. All muscle groups were intact with minimal swelling. The muscle appeared pink and contractile. The cubital and radial arteries were normally pulsating. The wound was left open, and progressive closure was planned depending on the patient’s progress from the vascular point of view of the surgery team.

On day 2, finger perfusion was impaired, and there was no visible pulse in the area of the mid-forearm. Diluted nitroglycerin was applied to the wound directly over the radial artery in an attempt to resolve the arterial spasms, and the carpal tunnel was decompressed.

The patient was kept in the Pediatric intensive care unit (PICU) for the following week on low-molecular-weight heparin (LMWH) and an intravenous antibiotic, nitroglycerine, and morphine infusion. Arm perfusion improved over the next few days. The Doppler signal started to improve on the radial arch and then later on the palmar arch and the fingers. On day 5, the patient was taken to the Operation Room (OR), and both forearm and wrist incisions were closed with no difficulty.

The patient was transferred to the regular ward on the day of surgery and was discharged on day 8 in good condition, with a palpable radial pulse and good triphasic Doppler signals on the fingers.

The patient was advised to continue LMWH for two more weeks, along with oral antibiotics and pain control medications. He was followed-up in the vascular outpatient clinic weekly for the following 4 weeks with normal wall-to-wall color flow and a triphasic waveform.

On day 17, a nerve conduction velocity (NCV) study showed reduced CMAP amplitudes for the left ulnar and radial nerves along with normal distal motor latencies, conduction velocities, and F-wave latencies.

The patient continued to complain of pain in the left shoulder and elbow, with tenderness, muscle spasms, left elbow flexor weakness, and a limited range of motion at the elbow and wrist joints. His motor function improved after he received ten sessions of physiotherapy over a period of 6 weeks.

The patient and his parents found the time to come to the hospital to thank the vascular surgery, pediatric intensive care, and physiotherapy teams after the patient’s complete recovery.

## Discussion

Children are more prone to jellyfish stings, as they stay and play in shallow water for a longer time, and their skin has less mechanical protection, with a thin epidermis and less hair coverage [9].

Reactions to jellyfish stings may vary from a mild skin rash to life-threatening symptoms. Species of jellyfish with lethal stings have tentacles studded with stinging nematocysts. During the stinging process, the nematocysts are released, and venom is discharged upon mechanosensory stimulation. The venom contains toxic peptides, phospholipase A, and histamine-liberating factors. Victims usually report an immediate prickling or burning sensation, pruritus, paraesthesia, and throbbing pain with radiation. The skin becomes reddened, darkened, edematous, and/or blistered. Neurologic, cardiovascular, respiratory, rheumatologic, gastrointestinal, renal, and ocular symptoms have been described with possible anaphylaxis [10].

Following a jellyfish sting, the skin should be decontaminated immediately with a generous application of vinegar, rubbing alcohol, baking soda, fresh lemon juice, or olive oil, together with shaving the skin to help remove the remaining nematocysts and effectively control the initial reaction. For the sting of the venomous box jellyfish, local application of heat (up to 45 °C) by immersion in hot water may be effective. Freshwater irrigation and vigorous rubbing lead to further stinging by adherent nematocysts and should be avoided. After decontamination, topical application of an anesthetic ointment (lidocaine, benzocaine), an antihistamine (diphenhydramine), or a glucocorticoid (hydrocortisone) may be helpful. Persistent severe pain after decontamination may be treated with narcotic analgesics. Muscle spasms may respond to diazepam given intravenously. An ovine-derived antivenom is available from Commonwealth Serum Laboratories for stings from the box jellyfish found in some countries.

Our patient was initially treated with pain medication and local creams and presented with limb-threatening complications.

**Fig. 4:**
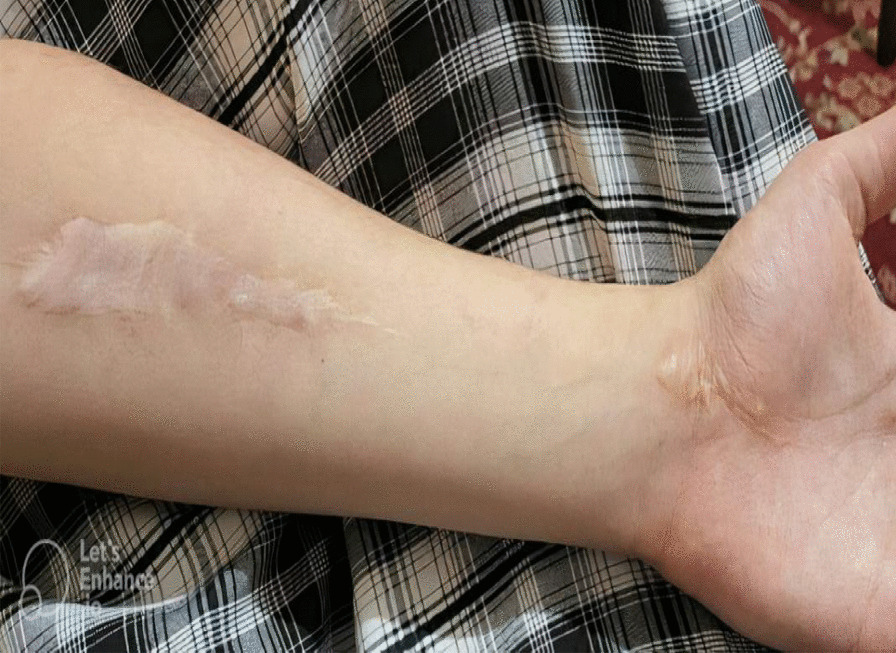
Arm appearance 6 weeks after surgery

He was treated with urgent fasciotomy to relieve vascular impairment associated with compartment syndrome, followed by medical treatment that included an intravenous antibiotic, nitroglycerine, and morphine infusion; tramadol; and subcutaneous (SC) calxane, and good functional outcomes were obtained (Fig. 4).

## Conclusion

The wide diversity of jellyfish species with venoms with different compositions of toxins and the variety of resulting symptoms make it almost impossible to define standard therapeutic interventions. Current therapy recommendations are mainly based on case reports.

Our patient presented to our pediatric emergency service with compartment syndrome that required urgent fasciotomy and local application of nitroglycerin, and a good outcome was achieved.

## Data Availability

Supporting data are available.
